# Collectanea

**Published:** 1827-11

**Authors:** 


### COLLECTANEA.

Floriferis ut apes in saltibus omnia libant,
Omnia nos, itidem, depascimur aurea dicta.

### ANATOMY.

*Structure of the Nerves.*—MM. Breschet and Raspail have examined the opinion of M. Bogros, who asserted that the nerves were of a tubular structure; and they have come to the conclusion, that the latter anatomist had been led into error by the facility with which injections penetrate the substance of nerves. *(Bulletin Univer.)*

*Abscess of the Liver bursting into the Pericardium.*—The following interesting case of pathological anatomy occurred last winter, in the dissecting-room of Jefferson College. The subject was a black female, of about thirty-five years. On opening the abdomen, there was discovered a prodigious abscess of the liver, occupying nearly the whole volume of that organ, and filled with an offensive pus, deeply coloured with bile and the dissolved substance of the organ, shreds of which were floating in the fluid. The colon was firmly adherent to the inferior surface of the liver, and ulceration was rapidly eroding the septum thus formed between the cavity of the colon and that of the abscess. But what was most remarkable, and I believe unique, (though perhaps I mistake,) the liver had formed a similar adhesion to the diaphragm on the left, beneath the heart. Through this adhesion an opening had been made into the cavity of the pericardium, and this cavity was filled with at least two pints of the same dark sanies which occupied the liver. The fluid must have occupied the pericardium for a considerable time, since that membrane was distended much beyond its natural capacity; and since, also, the surface of the heart was thickly studded with short tooth-like processes of concrete albuminous exudation, giving it an appearance which Laennec has compared to that produced "by the sudden separation of two pieces of slab joined by a pretty thick layer of butter."

Abscesses of the pericardium, even containing a much greater quantity of matter, are not very uncommon, but it is very rare that nature, as in this instance, foils her own recuperative efforts by conveying, from another part, the contents of an abscess into this cavity, where it must necessarily prove fatal.

The previous history of this case could not be obtained. *(Philadelphia Monthly Journal of Medicine and Surgery.)*

### PHYSIOLOGY.

*On the Origin of the Power of suspending Respiration, possessed by Aquatic Mammalia and Birds.* By Lawrence Edmonston, Esq. [From the *Philosophical Magazine.*] Very curious

To determine the nature and conditions of that power which certain species of aquatic animals possess of suspending respiration for a considerable time, has long been an interesting object of physiological investigation; but the most accurate observations and ingenious experiments seem hitherto to have failed in removing the obscurity in which this singular phenomenon is involved.

Can this have arisen from the erroneous direction in which research has been conducted; or can it result from the insurmountable difficulty of the subject? Has the obvious suggestion been sufficiently attended to, that this anomaly of respiration may constitute one of those ultimate facts in the laws of vitality which, in its nature, may be independent of peculiarity of organisation?

We observe certain diving animals that breathe through lungs, as whales, seals, and water-birds, remain long under water at one time, and not merely in a state of quiescence, but often expending great muscular exertion. We know that a much shorter period than this, without respiring, must be necessarily and immediately fatal to land animals similarly formed; and we are then too hastily disposed to infer, that this singular faculty which aquatic animals possess must depend on some undetected peculiarity of structure. The problem was supposed to have advanced towards solution, when it was asserted that, in aquatic animals, the foramen ovale remained open, and that in them there was something analogous to fœtal circulation.

But, in the first place, it is unproved that in them the foramen ovale is oftener open than in land animals; and, in the numerous individuals of the two species of seal (*Phoca barbata* and *P. vitulina,*) which I have dissected, I have never found it open, but in fœtuses. But, secondly, though this were the fact, it affords no solution of the question,—of the faculty which these animals possess of supporting life, not merely like the fœtus, with the external senses dormant, and supplied with oxygenated blood through the medium of the mother, but in all the play and vigour of their faculties, without the action of the lungs or the access of oxygen. The foramen ovale remaining open may cause less blood to circulate through the lungs, and more to be thrown on other organs; but the fact by no means accounts for the animals being able to sustain vigorous and perfect life, while venous blood must be circulating through the brain, and the vital function of respiration totally interrupted. If it be conceded that provisions may be found, in the increased size of the liver, spleen, or veins of the abdominal viscera, for receiving an additional quantity of blood, diverted from the lungs during the suspension of respiration, still this points out only a change in the distribution of the circulating fluid.

It can hardly be assumed that, the moment the animal dives, those processes, which exhaust the blood of its oxygen, are interrupted; for still, at the moment of suspending respiration, much venous blood must necessarily be existing in the circulation. And can we suppose that, until respiration is renewed, the venous blood remains in the pulmonic system without being carried to the systemic heart? Such an hypothesis, not more visionary than some others, only, like them, multiplies difficulties: it supposes, for instance, a power of suspending the processes which exhaust the blood of those vivifying principles which it receives from respiration. It supposes the pulmonic and systemic hearts to be, not merely in function different, but in action separate from and independent of each other. It presumes that the left ventricle contracts on vacuity, or that the aorta and its tributaries can, at the will of the animal, become substitutes for the action of the heart.

Can we assume that some other organ is vicarious of the function of the lungs, analogous to what we observe to a certain extent to obtain in some secretory functions, as those of the skin and kidneys; or that the system enjoys the power of accumulating an additional stock of oxygen to supply its diving exigencies? Or can we imagine that the liver, or any other organ, deprives in a great measure the venous blood of those qualities which render it so deleterious to life in terrestrial animals, when carried through the arteries to the brain, as the experiments of Bichat especially, and of other eminent physiologists, luminously demonstrate? To all these suppositions the same general objection applies,—that they are not only entirely destitute of proof, and inadequate to the solution of the difficulty, but multiply others equally unaccountable. While, therefore, the most minute and laborious anatomy has failed to unravel any peculiarity of structure adequate to account for this phenomenon, the doubt naturally arises, whether, in every case of difference or modification of function, we are entitled to presume difference of structure; whether the faculty in question may not be one of those modifications of the vital principle independent of tangible peculiarity of organisation? And, under this impression, the hypothesis which appears to me to be the simplest, and to be supported by analogy, is, that the conditions of the nervous systems of aquatic animals are such that venous blood requires a much longer period to circulate through their brain than in that of land animals, to produce the same deleterious effects. Why should it be matter of surprise that the brain of aquatic animals should have peculiar relations to certain fluids circulating through it; or that their nervous system should be less or more susceptible of sedative or stimulating influence? Different animals, and even different organs in the same animals, are very differently affected by similar causes; and in such cases we do not always necessarily presume difference of structure. Is it even wonderful that the excitability of aquatic animals should be such as to render them unable permanently to bear the same highly oxygenised blood as land-animals; much in the same way as we suffer, and should at length die, by breathing air in which there is more than its usual proportion of oxygen? Different species of terrestrial animals require, *cæteris paribus*, unequal quantities of oxygen; and even different individuals of the same species, both in health and disease, are dissimilar in this respect.

Why may not the natural and healthy state of the blood in aquatic animals have a tendency to the venous, as that of terrestrial animals has to the arterial standard? And this, indeed, I believe to be the fact; for, in all amphibious mammalia and birds which I have examined, the blood has much more of the venous than arterial appearance. If this fact be established by a sufficiently ample induction, it would seem decisive of the question; and it would furnish a satisfactory reason why aquatic animals decline and at length die, when placed in situations where they are long deprived of the power and the necessity of diving.

I have also observed, that if seals, after remaining on the rocks for an hour or two, are killed before returning to the water, the blood is more florid than under opposite circumstances; and that, after long reposing on land, and then betaking themselves to their native element, they in no instance, when they first dive, remain so long under water as in most other occasions. These facts may be obviously accounted for by supposing that respiration in these situations being longer continued without interruption than usual, the blood had become more highly oxygenised; and that hence the brain, being temporarily more highly excited, could not at once bear so long a continuance of the venous influence. I have repeatedly ascertained that the dark venous blood of the seal, when exposed to oxygen, as speedily assumes the arterial hue as that of any other animal.

To a certain extent, also, this capacity of suspending respiration is under the influence of habit, as we see exemplified by those individuals, even of our own species, engaged in the pearl fisheries. And moreover, this faculty, even in aquatic animals, is limited, and differs in the different species; for beyond a certain period they, like land animals, are unable to remain under water. I have seen seals taken in a net, and, when not allowed to come to the surface to breathe, life became extinct generally in less than a quarter of an hour. In this case, it is true, the violent struggling to get loose would naturally abridge the period during which they could remain under water in a tranquil state, which I should believe to be somewhere about twenty minutes.

The young of the great seal, like the young of the other species of Phoca, is brought forth on land, and must remain there a month or six weeks before it can live in the water. If before this period it be thrown into the sea, it exhibits as much anxiety and awkwardness as a young dog, and cannot remain longer, without inconvenience, under water. The young of the common seal, on the contrary, follows the mother to the water immediately it is born, and swims and dives with ease and perfection; a difference so strongly marked, and so decidedly established, as, in my opinion, sufficient to constitute, among others, a most important feature of specific distinction between them.

With respect to whales, I have been often assured by intelligent Greenland shipmasters, that they have been frequently known to remain upwards of an hour under water; and this also accords with my own observation. The difference, therefore, in this faculty, which we remark in different kinds of aquatic animals, and between these and land animals, seems more to consist in degree than in kind.

If a land bird, as a crow or a pigeon, be thrown into the water, even while the head is kept up, it is as speedily *drowned* as a cormorant under it. For this fact we can give no other adequate solution, than by referring it to some peculiarity of the nervous system. It may, indeed, be argued that the cause of death in this instance results proximately from the spasmodic closure of the glottis, produced by the shock of immersion. But why should this occur in the land bird more than in the cormorant?

Not only is the quality but also the quantity of blood in aquatic animals different from that in land animals. In the former, the quantity is much greater in proportion to their size. In whales and seals, for instance, it is excessive; and their capacity of great and continued muscular exertion is also strikingly conspicuous. Can this depend chiefly on the different quality or increased quantity of the vital fluid; or on some particular condition of the nervous energy?

On a review of what I have here advanced on this subject, it appears to me that we may be justified in assuming that the nervous system of aquatic animals breathing through lungs is so constituted, that the venous blood requires a much longer period to circulate in the brain before it produces death than in land animals. That the natural and healthy state of their blood is *sub-arterial;* and that it is not necessary, in accounting for the superior power which they possess of suspending respiration, to presume any peculiarity of organisation.

These views I have long entertained; and they are founded on an ample experience of many years, both as a zoologist and a sportsman, in situations peculiarly favourable for accurate observation on the economy and structure of aquatic animals.

### PATHOLOGY.

*Piece of Iron in the Chest for Fourteen Years.*—William Hunt, aged forty-four, was admitted on board the Seaman's Hospital, on the 23d February, 1826, with severe inflammation of the contents of the thorax, and died in a few hours. On admission, he stated that in 1812 he received a wound in his left side from a musket-ball, which was still in his chest, and that since that period he had been subject to violent inflammations after exposure to wet or cold. The present attack came on a few days ago, after arriving from sea, where he had been much exposed to cold.

*Sectio cadaveris.*—On opening the thorax, the attention was directed to the left side, principally for the reason that there was a very obvious depression observable on the surface of the third rib. Continuing the examination, which was pursued with extreme caution, a cyst was seen, which was found to contain a piece of iron hoop about an inch in length, and of the form of a crescent. The lung of that side was completely hepatised. The upper part of the trachea and bronchia contained pus. The other lung was comparatively sound, inasmuch as the bronchia of that side did not contain pus; neither was that lung hepatised. There were slight adhesions. (*Medico-Chirurg. Transact.*)

---

*Inflammation of the Iliac and Femoral Veins;* by Dr. Forbes.—Francis Merchant, æt. twenty-six, a journeyman printer of intemperate habits, was conceived to be labouring under pulmonary disease, which was accompanied with swelling of both ankles.

The swelling of the right ankle, however, gradually subsided, whilst that of the left, about a month previous to his decease, extended upwards, and increased, attended with pain about the ankle, calf of the leg, knee, ham, groin, and lower part of the abdomen. The pain in the knee was so considerable, in the early stage, as particularly to engage the attention of the mistress of the house in which he lodged, who thought he must have hurt the knee. Together with these symptoms, the whole limb became hotter than the other, and tender upon pressure.

When I first saw this patient on the 31st of October, a week before his death, I found him in the last stage of pulmonary consumption, having cough, purulent expectoration, diarrhœa, and considerable emaciation. The whole of the left lower extremity was now enlarged to double its natural size, the swelling extending from the foot to the crural arch. Everywhere it pitted upon pressure, but it was no longer painful when touched, except at the groin, where uneasiness was still experienced.

The colour of the skin of the limb was whiter than natural, and presented an appearance perfectly similar to that observed in common anasarca. The subcutaneous veins above the ankle were distinctly marked, being distended with blood. The patient, who had lost all power of motion in the limb, died on the 8th of November.

On the following day Mr. Grainger examined the body, in the presence of Dr. Macann, several other gentlemen, and myself. An incision was made along the inner part of the thigh, commencing at Poupart's ligament, and continuing below the knee. On exposing the vena saphæna major and its branches, these veins were observed very much distended; and, being laid open, were found filled with coagulated blood, but there were no appearances of disease of their coats. The cellular tissue of the whole limb was very much infiltrated with a limpid fluid, a quantity of which escaped from the incision of the integuments. The lymphatic vessels were quite healthy; and the lymphatic glands in the groin were rather enlarged.

The deep-seated veins were next examined, and presented a similar appearance to that of the superficial veins; the femoral vein being tense, and filled with coagulated blood.

The dissection was continued so as to expose the left iliac veins. The trunk of the external and common iliac vessels were found even more distended than the femoral vein. The common iliac vein had an unnatural colour, approaching to a greenish hue ; and it seemed as large as the inferior cava. The distention of this vessel suddenly terminated at that part where it is united with the right common iliac vein. The left internal iliac vein was filled with blood for about two inches of its course, and the rest of it appeared natural.

The femoral and iliac veins on the right side were nearly empty, and quite healthy in their appearance, forming a strong contrast, when compared with the gorged and distended state of the veins on the left side.

On laying open the upper part of the femoral vein, as well as the external and common iliac veins, they were found filled with a coagulum, of much firmer consistence than is usually met with in healthy veins; and had much of the fibrous appearance of the blood which is found contained in aneurismal sacs. On separating this coagulum, a thin but distinct membranous layer was seen adherent to the internal coat of the vein, and it required some force to separate them. The femoral vein, which was examined as far down the limb as the triceps muscle, was found in the same condition.

The morbid appearances observed in this instance were very similar to those which have been described by Dr. Davis, in his paper on Phlegmasia Dolens, inserted in the twelfth volume of the Transactions of this Society. Had the subject of the disease been a woman in the puerperal state, would it not have been considered phlegmasia dolens?

Morgagni, letter 56, art. 10, in treating of disease of the iliac veins, relates the case of a woman whose left limb was œdematous; and, upon examining the body after death, he found the left iliac vein filled with a coagulum, as he terms it, of a polypous nature, the canal being quite obliterated by it. The crural vein was at least a third part narrower, and appeared to have been in that state for a considerable time.

I lately attended a woman, sixty-three years of age, the mother of a family, who, several months previous to my seeing her, was attacked with swelling and lancinating pain in the right foot, which extended upwards to the groin, where, as well as in the knee and in the calf of the leg, she experienced considerable pain.

On my first visiting this woman, on the 20th of December, I found the whole limb, from the foot to the hip, swelled to double its natural size, colourless, and pitting upon pressure. The urine was secreted in natural quantity. Cupping and blistering, in the course of the femoral vein, were had recourse to; which treatment was followed by a very considerable reduction in the swelling. A bandage was afterwards applied. The leg and thigh are now of their natural size, but remain without power; the foot is still slightly œdematous. (*Ibid.*)

*Pathology of Chorea.*—M. Serres having remarked, in the opening of the bodies of those who died from chorea, a tendency to inflammation of the corpora quadrigemina, was induced to experiment on animals, by wounding them in these parts, and which he observed to produce phenomena similar to those of chorea. M. S. has observed that chorea patients always complained of pain at the posterior and inferior part of the cranium: he has, in consequence, directed the application of leeches to that quarter in such cases, and with success. (*Revue Med.*)

---

*Case of Hydrophobia, as communicated in a Letter to* J. A. Maxwell, M.D. *Superintending Surgeon at Bombay;* by R. H. Kennedy, M.D.

Mr. M., assistant surgeon, was bitten by one of his own dogs on the 21st March, and the animal being then tied up, made its escape the following day, and was never seen afterwards: most probably was destroyed in the bazar, and the fact concealed by those who did it. Lieut. S. says, he warned poor M. of the state the creature was in several days before the lamentable accident, during which interval the brute bit every thing it saw, even the goats. M., however, most unaccountably neglected these warnings, and attached no importance to the appearance of madness which the animal evinced.

Until Tuesday the 2d, no disagreeable symptoms showed themselves. The wound had healed at once, being very slight; but, having occurred on the bare skin, on the hand, the inoculation of the poison should have been looked for in its fullest malignity; but unhappily no precaution, beyond washing, seems to have been thought necessary. On Tuesday morning, when feeding some tame deer he kept, one of them bounded at him, and with its horn struck him on the fore-arm, midway between the wrist and elbow: the blow was so severe, that his first feeling impressed him with the idea that the bones were fractured. The severity of the contusion brought on immediately great pain and swelling of the limb; and, it being the same on which he had been bitten, the acute pain seemed to concentrate about the faint scar of the old wound. On Wednesday afternoon, the 3d, I wrote to invite him to dine with me on Saturday the 6th; to which he replied, accepting my invitation, but added that he had a pain in his arm, which he hoped might prove nothing worse than rheumatism. Feeling himself worse in the evening, he sent for his neighbour, Mr. M'Morris, to whom he detailed his symptoms.

About eleven that night, I received a note from Mr. M'M., stating what he had seen and heard from M., and distinctly expressing his uneasiness for the result; and, after mentioning that he had given ten grains of calomel and fifteen of extract of colocynth, asking what opinion I felt disposed to form, or what treatment I would join him in adopting. I suggested bleeding and opium; but, reverting to the late melancholy case of hydrophobia at Dapoly, (the hearing of which I knew had greatly distressed Mr. M.) I hoped that the local pain on the site of the bite, as greater there than in any other part of the swollen limb, might be fancy and nervousness, arising from the awful consequences apprehended, and the dreadful agitation likely to follow so appalling an apprehension.

I visited the poor fellow at daylight, and found him in better spirits than I had expected. His arm was swollen to the shoulder; the scar from the bite was scarcely to be recognised, though it indicated the seat of a pain resembling a thorn being buried in the flesh; but the blow from the deer was evident by a blue and yellow bruise. As this had therefore certainly been very severe, I hoped it might account for the inflammation of the arm and hand, and the pain now rising past the axilla to the chest. He had wrapped the arm in cloths moistened with Goulard's lotion, which he was applying himself, daubing the liquid unconcernedly over the bandage. As the nervous agitation was, however, very great, the pulse full, and the constitution plethoric, I recommended bleeding as a general treatment, and camphorated anodyne spirituous fomentations locally. The purge having no effect, M'M. repeated the dose in the forenoon, and took away twenty-four ounces of blood.

I saw him the second time in the evening, when he was sitting at his door. He was dejected and weak, but thought his arm relieved. Nothing hydrophobic appeared to attract attention. The pulse indicated no disorder beyond the reduced strength and frequency to be expected after phlebotomy. The two purges not having operated, M'M. administered the same dose, being the third, in the evening.

I saw him again the next morning, (Friday the 5th,) about sunrise. The purgatives had acted most powerfully. He had spent a miserable night, and seemed much disordered at stomach, with constant nausea, and vomiting whatever he swallowed. The inflammation of the limb below the bruise inflicted by the deer seemed abated, and he could extend or close the fingers; but the pain in the axilla and about the thorax continued spreading and acute. He had now a distinct and distressing feeling of the nature of his disease, and mentioned to me his intentions respecting his worldly affairs; and, when I endeavoured to comfort him, he interrupted me by saying, with tears, that a peculiar difficulty of swallowing now began to force itself on his attention. I immediately examined his throat, and seeing a bright florid redness and puffiness about the fauces, and a slight enlargement of the tonsils, and a ruddy swollen, rounded, edgelike appearance of the gums round the bases of the teeth, I hoped the thirty grains of calomel might explain this inconvenience, the mention of which relieved him for a short time of much mental agony; but I could not help feeling pained to observe a singular fact, that his breath was unaffected by the mercury, and perfectly pure; whereas in health, ever since I have known him, it had always been most offensively fetid. The symptoms continued rapidly progressive through the day, during which M'M. was constantly with him. He wrote me about noon, that debility with cold sweats was too marked a feature of the case to admit of further bleeding.

I visited him again early in the evening, when I found the hydrophobic convulsion the prominent character of this disease, accompanied with unremitting vomiting. I then suggested to M'M. that the singular and complicated manner in which the attack had commenced still left a hope, or rather the shadow of a hope, that this nervous agitation might be alarm carried to mania, when operating on the brain and constitution, in conjunction with disorder of stomach, deranged to the utmost by three such powerful mercurial drastic purges, and a throat apparently swollen by them; and I mentioned two cases I had seen in London, where the hydrophobic horror was more conspicuous than in the case before us, and eventually proved hypochondriacal agitation, following wounds from dogs not mad, and in which cases the patients recovered: I therefore begged him to try opium, in such doses as the excessive agitation would alone justify. Accordingly drs. ii. of Tinct. Opii were immediately given, and at once vomited up; the same, repeated in an hour, was again rejected. A pill of opium grs. iii. was swallowed and retained. We were with him at ten P.M. and introduced an enema of drs. iv. of Tinct. Opii, which M'M. repeated in the night; and a large blister was applied to the scrobiculus cordis, to relieve, if possible, the exhausting sickness.

M'M. staid the night with him, a duty in which I could not assist him, being myself summoned to another patient, a lady, on whom I was in attendance the greater part of the night. This circumstance, and urgent duty in the morning, prevented my paying my usual early visit; but I was gratified to receive a note from M'M. saying that he at last indulged hopes that poor M. was certainly better, and had been able to swallow without convulsions during the early morning hours.

I saw the poor fellow before noon, and remained upwards of an hour with them. The slight appearance of amendment had soon ceased to deceive, and the hydrophobic horror was now fully and incontestibly developed, and the patient was rapidly sinking under the violence of the spasms, which appeared to threaten momentary suffocation. His skin was deathly cold and clammy, his face gaunt and pale; but his lips and tongue were of a carmine red hue; his eyes wild and prominent, but not at all bloodshot; and all his appearance marked his case hopeless. He further complained of a tingling sensation in the glans penis, and involuntary micturition; and when we begged him not to attempt every moment to swallow, thereby bringing on the convulsions, he said that, if he allowed his tongue to be dry, it felt as if it rolled itself up, and rushed backward with a sense of suffocation: a most extraordinary symptom, indicative of the dreadfully convulsed and agitated state of the muscles of deglutition.

As medical assistance now ceased to be of avail, I left him to beg the Rev. Mr. J., who had already been with him, to administer religious comfort, to visit him again, and to get his signature to a will: for, as he told both M'M. and myself his testamentary intentions, we preferred having this upon paper, to its being a nuncupative arrangement. Previously, however, to my departure, he insisted on giving me a proof of his resoluteness in conquering the mania, and, calling for a glass of thin arrow-root, he forced down, with the most distressing struggle, about four ounces; an effort immediately followed by the most convulsive retching. I never saw a sight more afflicting.

When Mr. J. offered to make a will for him, he was pleased with the proposal, attended to the reading of the paper, and directed an alteration as to the mode of remitting the proceeds of his property from that which had been first written. He ordered a donation of a month's pay to each of his servants; mentioned some arrears of pay due to himself; and noticed that a certain horse in his stable was not his own property, but left in his charge for sale. In signing the will, being in the interval betwixt the convulsions, he stood up to a writing table, and wrote his name at full length, subscribing his army rank and the number of his regiment, as to an official paper; and actually jested at his occasional control over the mania, and his strength and self-possession. He had not, however, a long repose; the disease was making horrible progress. He began about four to foam at the mouth, the liquid rushing forth with his convulsive respiration, gurgling in frothy masses, or running in a continued stream to the ground. During this period I was not with him; but M'M. says, he kept his sense of passing occurrences and recollection of individuals to the last, and finally expired quietly and without convulsion at five. He scarcely expired ere the florid red hue of his lips changed to blackness, and his countenance altered considerably, appearing swollen and bruised.

The curious features of this case appear to me, the complicated and perplexing manner of its accession from the bruise in the fore-arm, awakening the dormant hydrophobic venom lodged in the hand. Are we to suppose that excision up to that moment would have removed the poison, so as to have saved the patient? Or might we argue on a possibility that, but for some such excitement, it may remain an indefinite period, until gradually exhausted, and discharged by sensible or insensible perspiration?

The next is the perfect self-possession he enjoyed to the last moment, and which he evinced in perpetual efforts to resist the convulsive shocks experienced in his attempts to swallow. He seemed unceasingly desirous of trying it as an experiment whether he were better or worse. On the night before his death, he would say, "I can wash my hands, but not my face:" and then calling for water, would dip his hands in it, and make painful, but unavailing endeavours to raise a moistened towel to his forehead. His description of the spasm was in these words: "You know the feeling of losing one's breath on plunging suddenly into a cold bath? My sensation is exactly the very excess of that kind of shock, coming on with an indescribable chilly thrill through me, and acting with such violence as if the power of respiration were lost altogether." Three hours before death, he observed that all his evils but the suffocation had left him. His hand and arm were quite free from pain, and he moved the fingers nimbly. He added that he "could drink in the dark." In fact, he never yielded to the disease; his spirit remained unshaken, until nature sank exhausted.

His manner and temper, on the approach of death, were what both had been through life, mild and kind: no frowardness, no harshness of repining, no unmanly lamentation or complaint. He caressed each friendly hand that was extended to him, and simply stated that his sufferings were extreme. To us he declared himself patient to endure whatever we wished, and even suggested tracheotomy,* an operation which I never saw performed, and which we declined, as well from a consciousness of its utter inutility in his state, as from a conviction that the exuding of a single drop of blood inwards would suffocate him at once. In a case like this, the medical man's sufferings are tenfold more miserably acute than another's would be; his professional knowledge of every symptom as it appears fixes his attention inwardly, and the dread of their approach too often accelerates their progress; and the familiarity with their character and tendency, at least must add virulence to their malignity.

I know nothing further that could have been done. He could not have been bled more; he would have fainted and died. The incessant vomiting rendered the exhibition of medicine horribly painful: to be swallowed merely, to be immediately rejected. The enema was used to the utmost extent it could be A larger blister could scarcely have been attempted; and opiate embrocations were kept by flannel bands permanently applied to his throat. Nor ought I to omit mentioning, in the terms it deserves, the unwearied humanity, anxious thought, and constant presence of mind shown by Mr. M'M., who left him as little as his other duties would allow, and has raised himself in the respect and esteem of all here by such exemplary kindness.

* In some observations made by Mr. Mayo, at the Westminster Medical Society last season, he suggested the performance of tracheotomy as a fair experiment in Hydrophobia.

### PRACTICAL MEDICINE.

*Sulphate of Copper in Diarrhœa.*—The volume of the *Medico-Chirurgical Transactions* just published contains some important practical remarks on this subject, by Dr. ELLIOTSON. We subjoin the most interesting of these.

On Thursday, October 13th, 1824, I admitted into St. Thomas's Hospital an Irishman, named Garret Welsh, twenty-four years of age, who had been labouring under diarrhœa for three months.

The abdomen was tender on pressure, the pulse quick, the skin hot, and the tongue foul and dry. The motions were yellowish and watery. I ordered a very large blister to be applied over the abdomen, half a grain of opium to be taken night and morning, with chalk mixture, and the diet to consist of milk. In a few days the tenderness was much diminished, but the diarrhœa continued. Powdered catechu was added to the mixture, and taken every six hours; and the blister was repeated, but without good effect. Infusion of galls was now made the vehicle of the catechu, and instead of the solid opium, taken latterly in the dose of one grain night and morning, f℈j. of the tincture was given in such astringent draught, a third blister was applied, and, as considerable debility and emaciation had taken place, animal diet was substituted for the milk.

The 30th of November had now arrived, and, though there was no pain or tenderness, the diarrhœa was as profuse as ever, and the man appeared likely to sink. Mr. Henry South, one of the pupils, mentioned to me that two cases of the successful exhibition of sulphate of copper and opium in diarrhœa had recently been related in the Physical Society of Guy's Hospital, and I instantly resolved to profit by his information, and prescribed half a grain of the sulphate of copper twice a-day, with two grains of opium, so that the quantity of the latter might be much the same as what had previously been taken in the twenty-four hours. In a few days the disease was evidently less severe, and in eleven from the commencement of the use of the copper was so much abated, and the man's appearance and strength so much improved, that I ventured to diminish the opium to one grain; increasing, however, the dose of the copper to one grain, lest the disease should gain ground again, and that I might fully satisfy myself which of the remedies had the greater share in producing the benefit. In a week more the disease had so diminished that I ventured to omit the opium; and in another the man felt so strong, and found his disease so inconsiderable, that he would stay no longer in the hospital, and was made an out-patient.

This case made a great impression upon me, because two powerful astringents in common use, combined with opium, had previously failed, and the benefit clearly arose from the sulphate of copper, and not from the opium, which I exhibited with it solely for the purpose of preventing the acrid quality of the salt from counteracting its astringent effects upon the intestines.

On December 8th, 1825, I admitted a sailor, named Peter Hurly, aged forty-eight, who had been affected many weeks with the form of diarrhœa so accurately described by Dr. BAILLIE, in the fifth volume of the Transactions of the College of Physicians. The description is preceded by the remark, that it "is not very generally known, and is almost constantly fatal." Dr. Baillie states, that it occurs more frequently in men than in women, and generally in persons who have long resided in hot climates; that the stools are very copious and numerous, pale, like a mixture of water and lime, frothy, and often of a sour smell; but, if they acquire the consistence of pudding, they are still pale; and if they become figured and dark, the colour is rarely so deep as in health, and they soon again become white and frothy; that the body is thin, and the countenance sallow; that there is no tumefaction or pain of the abdomen; and that, though the disease may continue several years, and be occasionally less severe, it almost constantly in the end exhausts the constitution.

The present patient was a *man*, and had lived much in *hot* climates; was *thin* and *sallow*, and had gradually lost his flesh and strength; his motions very frequent and *copious;* liquid, *white* and *frothy;* he had *no swelling* of the abdomen, *nor tormina, nor pain upon pressure.*

I ordered half a grain of sulphate of copper, with one grain of opium, to be taken twice a-day; and his diet to consist of milk, arrow-root, strong beef-tea, and a little wine. In four days the dose was increased to one grain. He had not taken this quantity above five days before the stools were reduced to two or three in the twenty-four hours, and they were never afterwards more numerous, except for one day, probably from some accidental circumstance. In a few more days they became thicker, and from that time there was generally but one in the twenty-four hours. On the 3d of January, rather less than four weeks from his admission, they assumed a deep healthy yellow,—an occurrence rarely, if ever, witnessed by Dr. Baillie,—and they continued of this colour. They, however, became liquid again, and I augmented the dose to one grain and a half; and this not rendering them consistent, I augmented the dose, after eleven days, to two grains. Some pain was now felt in the epigastrium, and a blister was applied without benefit; and, as it appeared to arise from the acrimony of the salt, the dose of opium was made gr. jss. with the effect of removing it entirely. The man found himself so strong that he desired to be discharged on the 2d of February. He had gained flesh, and now passed but one motion in the twenty-four hours, solid, and of a healthy appearance. He took with him a supply of medicines for a fortnight.

[Numerous other cases are added in illustration; after which, Dr. Elliotson continues:]

I could greatly extend this list, but the narration of cases is always tedious, especially when they are so similar, as such must necessarily be to each other; and the preceding sufficiently illustrate the power of the remedy. I will therefore content myself with mentioning the general results of my experience during the last two years and a half in St. Thomas's Hospital, where a great number of sailors are admitted labouring under intestinal affections consequent upon dysentery in hot climates.

I am satisfied that the sulphate of copper is superior to every other astringent in chronic diarrhœa; that it will cure the disease more quickly than any other, and often when all others fail.

Three grains three times a-day is the largest quantity I have prescribed; but this I have frequently given. The dose usually borne and required varies from a grain and a half to three grains. It may be taken for an indefinite time without any fear of constitutional ill effects.

I had two very severe cases, in which the quantity of blood and matter in the evacuations, and the wretched appearance of the countenance, rendered the existence of great disease of the inner surface of the intestines probable, and which would most likely have proved fatal but for the remedy. It arrested the complaint after a time; but, such had been the severity of the disease, and so much reason was there for apprehending mesenteric affection, and thickening of all the substance of the intestines, that, if it was omitted for a week, the diarrhœa in some measure returned. These patients took it between three and four months, in doses of two or three grains three times a-day, with no other effect than that of controlling the disease, and improving the appearance in a degree which surprised every one.

It certainly has a tendency to produce vomiting and griping. On these accounts I have always combined it with opium, and never, but in the first case, and then only for a week, and, at the cessation of the disease, ventured to exhibit it alone. No inconvenience resulted, and I have generally found a grain and a half of opium sufficient to prevent even three grains from griping in the least. Notwithstanding the unquestionable co-operation of the opium with the sulphate, the far superior share of the salt in curing the disease has been repeatedly proved by the previous failure of opium alone, or combined with other astringents, and by the dose of opium being actually diminished when the copper was superadded.

The disposition to occasion sickness is much diminished by administering it, like all medicines inclined to disagree with the stomach, and intended to pass the organ without sensible effect, in the form of pill, and after food has been taken. A dose of it, as well as of oxymuriate of mercury or tartrite of antimony, which would cause sickness if taken in a liquid form or before breakfast, will be borne perfectly well, and frequently indeed a much larger dose, if taken in the form of pill and after a meal.

In thus recommending a medicine for a purpose which, as far as I know, has not been mentioned by any author, I am anxious, as upon every such occasion, however great its excellence, not to excite too high an expectation,—not to appear pretending that it is universally applicable, and that it never fails. In cases only in which astringents are proper, is it proper; and although ulceration of the inner coat of the intestines, and very extensive ulceration, will heal, and the sulphate of copper will contribute to the cure more than any other medicine, the mischief may be irremediable by any measures: and, on the other hand, chronic diarrhœa is frequently kept up by the state of mind, by the mode of living, or by the residence. No medicine can alter these, and I knew two examples of the failure of it and all other remedies, when a removal from town cured the disease in a few days, and a return to the metropolis was invariably followed by a return of the diarrhœa.

---

*Muriate of Iron recommended in Softening of the Stomach.*—In the *Heidelberg Klin. Annalen*, Dr. de Pommer has related some cases of supposed softening of the stomach in infants, a disease to which the attention of the profession has recently been called, (see the paper of Dr. Gairdner, in the Medico-Chirurgical Transactions of Edinburgh, &c.) in which he administered the muriate of iron with apparent advantage. We say supposed cases, because the actual condition of the stomach in cases terminating favourably must remain matter of conjecture, and all that we can venture to affirm is, that the symptoms of the children who survived were very similar to those in the children who perished, and in whom the coats of the stomach were found softened. In the cases which did well under the muriate of iron, there was constant vomiting, with frequent, loose, fetid stools; moaning, and anxiety of countenance; but no tension of the abdomen, or tenderness on pressure. The following is the prescription recommended:—℞. Rad. Alth. ℥ij.; Coq. c. aq. fontan. q.s.; Colal. ℥ij.; adde Pulv. Gum. Arab. ʒij.; Ferri Muriat. ℈jss.; Syrup. Alth. ʒvj. M. S. Of this two tea-spoonsful are given every hour, care being taken to shake the mixture each time.

---

*Treatment of Inflammation by Tartarised Antimony.*—This practice appears to gain ground more on the continent than in this country: we use antimony, indeed, very freely in inflammatory affections, but always as auxiliary to bleeding. We have often heard it remarked, with reference to this subject, that treatment which might succeed in Italy would not answer in this country; but we find, from the testimony of Dr. Bang (*Bibliothek für Leger*), that it has proved successful in the north of Europe. Fifty-four patients were admitted at the Royal Hospital at Copenhagen with inflammatory affections of the chest. Forty-five of these had no other medicine besides the tartarised antimony; seven had other remedies in addition: all recovered except two. It is proper to state, however, as in our opinion materially affecting the conclusions to be drawn from the result of these cases, that the greater number were bled once before the remedy was begun.

---

*Application of Medicines by the Skin.*—Dr. Martin, jun. has been lately occupied in experimenting on what the French call "la méthode endermique" of exhibiting medicines; and, in the Number of the *Revue Medicale* for September, he has published a Memoir on the Exhibition of the Sulphate of Quinine by the Skin in Intermittent Fevers. He gives six cases in which the remedy was thus administered, in all of which (except one, in which it was found necessary to give the medicine internally,) it proved successful. Dr. M. considers the question of the absorbing powers of the skin as set at rest by the experiments of Bichat, Pinel, Chaussier, Alibert, and Dumeril. But, allowing a doubt to exist as to the power of absorbing where substances are simply applied to the skin, it does not apply here, as in this method the epidermis is previously removed by a blister. "The skin, deprived of its epidermis," says Dr. Martin, "possesses a power of absorbing so great and so manifest, that it admits not of doubt. Who does not know the terrible effects of rabies, and the venom of serpents, thus introduced into the body? Dr. Lesieurs is the first who proposed this function of the skin as a new method of giving medicines; and wished to assure myself of the results of a method which appeared likely to be so useful." The method he uses will be best shown by transcribing one of his cases.

Claire Bels, ætat. thirty-four, was seized, in the beginning of September, 1826, with remittent fever, which was stopped in a month by means of the sulphate of quina, given internally. Fifteen days after, a quotidian intermittent showed itself. The fever, which at first was slight, becoming severe, she entered the Hôtel Dieu, December 18th, with the following symptoms:—Face flushed, heat of skin, slight moisture; pulse strong, bounding, and frequent; tongue red and dry; frequent cough; pain in the chest, increased on coughing; slight headache; the other functions natural.—She was bled to eight ounces, and ordered pectoral medicine, with low diet.

19th.—A return of fever. A blister was applied to the arm.

20th.—Perfectly calm. The blister rose well. Six grains of Sulphate of

Quina in powder was sprinkled on the blister, and the wound covered with beet-leaves.—At noon, the paroxysm came on as before. Blister very painful.

21st.—Great pain in the arm, which is swollen and red. Six grains more of the Quina were put on the wound. No fever to-day.—In the evening, great pain from the wound, to which a poultice of linseed-meal was applied.

22d.—A bad night; much suffering; no fever during the day. Continue the cataplasm.

23d.—A quiet night; scarcely any pain, and no stiffness of the arms. No return of fever.

24th.—The patient is doing well. Slight pain from the blistered part; good suppuration. No return of fever.

26th.—The wound is nearly healed: scarcely any pain; no fever.

31st.—The patient has quite recovered, and has been dismissed cured.

Dr. M. soon observed that, by mixing the quina with the cerate with which the blister was dressed, the violent affects of the salt on the wound were moderated, and its absorption not at all retarded.

In enumerating the advantages of this mode of administering the quina, Dr. M. remarks that patients who cannot or will not take the quantity of medicine necessary for their cure, may in this way be brought under its influence, without their being aware of it. In children also, where it is sometimes nearly impossible to give medicine internally, this mode may be advantageous. In some cases of intermittent fever, where congestions or inflammation of the stomach or intestines prevent the internal exhibition of quina, this new method would answer. Sometimes also the medicine runs off immediately by the bowels, and in this way the first doses are entirely lost: this is avoided in giving it through the medium of the skin. Another advantage pointed out is, that there is no occasion to wait for the remission of the paroxysm, but it may be applied at any period of the fever. It appears also that, by being carried into the blood without alteration, much smaller doses of it will stop the course of the disease than when given by the stomach. The quantity of the sulphate necessary to stop the fever, when taken internally, is from six to twelve grains; when applied to the skin, about four grains will suffice in mild cases.

The only objection to this plan appears to be, that a blister becomes necessary.

Dr. M. draws the following conclusions:—1st. That the sulphate of quina, applied to the dermis, denuded by means of a blister, stops the progress of intermittent fever, when nothing is opposed to its absorption.

2d. This salt operates in these diseases by a general and specific, and not by revulsive or any local action.

3d. The absorption is very rapid.

4th. The salt thus absorbed suffices in smaller doses than when given by the stomach.

5th. The sulphate applied to the skin in powder causes great inflammation.

6th. The sulphate mixed with cerate causes scarcely any pain or inflammation.

7th. This medicine, valuable for administering the quina to certain patients in simple intermittents, may become a great resource in certain cases of dangerous and obstinate fevers.

### SURGERY.

The following observations are from a Clinical Lecture, delivered by Dr. Ballingall, at the conclusion of his summer course, 1827:—

*Fractures of the Thigh.*—It would ill become me, gentlemen, to underrate the importance of the discussion about fractures of the neck of the thigh-bone, considering the distinguished surgeons who have taken part in it; but while several points connected with this controversy are still undecided, I must, *pendente lite*, be permitted to solicit your attention to a division of fractures of the upper part of the thigh-bone capable of immediate practical application. I would divide these fractures into such as occur in that part of the bone above the muscular insertions, and such as occur at or below the point at which these muscular insertions commence: that is, into fractures above the trochanters; fractures passing through these processes; and fractures immediately below them,—of each of which there are numerous varieties.

In the first case, it is obvious that we have no command over the head and neck of the bone, except through the medium of its ligamentous connexions with the pelvis, and every contrivance calculated to ensure the successful treatment of this accident, must have in view the fixture of the pelvis, as well as of the thigh-bone. The limb may be placed either in the bent or extended posture; but the former I consider the best wherever we have an apparatus sufficiently perfect for keeping the parts in apposition.

In the second case, that of fracture through the trochanters, we have both portions of the bone acted upon, and sometimes rotated in opposite directions by the muscles implanted into them; and hence we are enabled to explain the fracture of this part of the bone with inversion of the toes; an occurrence which was long ago noticed by Paré and Petit, but of which the true explanation has only recently been given by Mr. Guthrie, of London, and Mr. Syme, of this city, each of whom has met with an instance of this variety. Of its treatment I speak with great diffidence, because I speak without any personal experience; but I am disposed to think that, in most cases, the tendency of the limb to deviate from the natural position will be best counteracted by keeping it in the extended posture by means of Boyer's, Desault's, or Hagedorn's apparatus; the merits and defects of each of which I endeavoured to explain to you.

In the third case, that of fracture immediately below the trochanters, the limb ought, I think, uniformly to be placed in the bent position. It has been well observed, that in this instance no modification of the extended posture will answer; for, supposing the patient placed on his back, the powerful action of the psoas magnus and iliacus internus muscles, inserted into the trochanter minor, will raise or bend the upper fragment of the bone upon the pelvis, while it is obvious that all attempts to extend, to level, or to depress the inferior fragment of the bone will tend to separate the broken ends from each other.

I would therefore press upon your attention these three varieties of this accident, as more capable of being recognised in practice, and better calculated to lead to a determinate and successful mode of treatment. In the first case, the position of the limb may be optional; in the second, I am inclined to consider the extended posture the best; and in the third, the bent position should invariably be adopted.

In each of the cases which have led to these observations, we had all the usual diagnostics of fracture above the trochanters,—fracture within the capsular ligaments; we had the limbs shortened in Sedler's case to the extent of an inch, and in Stevenson's case to the extent of an inch and a half,—the space between the trochanters and crests of the ilia in both cases diminished,—the trochanters rotating along with the shafts of the bones,—the eversion of the toes,—and the sense of crepitus on extending and rotating the limbs. They have both apparently obtained a pretty firm reunion, but whether of a bony nature or not it is impossible to say. These cases, you will observe, were both treated in the extended posture, as practised by the French surgeons; but this more from a sense of the imperfection of the apparatus which we possess for retaining them in the bent position, than from my own conviction of the advantages of the extended one. An early bias, derived perhaps from the study of Mr. Pott's writings on this subject, and the observation that the limbs naturally fall into the bent position during sleep, have made me long incline to the general adoption of the bent posture in the treatment of fractures of the lower extremities; and for maintaining this posture the best contrivances with which I am acquainted are the beds of Mr. Henry Earle and of Mr. Amesbury, the construction of which I endeavoured to explain to you by means of the plates attached to their respective works. For fractures in the lower part of the thigh or leg, I would recommend to your notice the splint contrived by Mr. Macintyre, of Newcastle, which you have frequently seen me use in this house, and which I consider one of the best modificatious of the double inclined plane.

---

*Calculus in the Perineum.*—In the case of Thomas Banks, also a patient of Dr. Hunter's, you saw a very extraordinary occurrence—the lodgment of a large-sized stone in the perineum, communicating with a small fistulous opening externally, but having no communication with the canal of the urethra. This patient was admitted on the 5th of April, and stated that, about eight years ago, a tumor formed in the seat of the opening in the perineum,—that this tumor burst spontaneously, and that the orifice had never since closed. He had never experienced any difficulty in voiding his water; none of it had ever escaped by the sinus; nor had his complaints ever debarred him from his usual avocations. In this case, the first impression was, that the hard body felt in the perineum was an exfoliation from the ischium; but Dr. Hunter, on introducing the probe, conceived it to be a calculus, immediately cut down upon it, and removed it by means of a pair of dressing forceps: the wound granulated kindly, and the patient was dismissed cured on the 30th of April.

This concretion, which weighed eighty-nine grains, was sent to Dr. Turner for analysis; and in a note from him, which I had the pleasnre of reading to you, it is stated to consist of "the carbonate and phosphate of lime, together with a little animal matter," thus differing, I believe, essentially in its composition from any of the numerous calculi from the human bladder which have been analysed by Marcet or others. Some concretions akin to this have, I think, been found in the bladders of the inferior animals; but, supposing the nucleus of this formation to have come from the patient's bladder, how and when did it escape from the urethra?—how did it increase to its present size?—how did the aperture in the urethra close over it? and how did all this take place without any impediment to the excretion of the patient's urine? From the difficulty in solving some of these questions, and from the constituent parts of this concretion, it can scarcely, perhaps, be thought to have originated in the bladder: but, as all this is matter of speculation unconnected with any surgical bleeding, I must hold myself excused from entering further into this extraordinary case. (*Ibid.*)

---

*Loss of a Portion of the Windpipe, without Loss of Voice.*—M. CLOQUET, at a late meeting of the Royal Academy of Medicine, "*presented*" a hair-dresser, who had cut his throat with a razor. The wound was transverse, and had divided the windpipe in such a manner that two of the rings were entirely detached at their anterior part, and were only retained behind by a portion of cellular membrane. They were removed; the lips of the wound brought together by suture and bandage, and cicatrisation took place without any fistula, although there was loss of substance, and the wound admitted the finger even when the head was bent. The voice was lost in the first instance, but afterwards returned, although it remained hoarse. Similar cases are mentioned by LARREY.

---

*Application of the Hydrocyanic Acid in Cases of Herpes.*—In a recent Number of RUST's Magazine, some cases are recorded of herpetic diseases, which were relieved by the external use of this remedy. A drachm and a half of the acid mixed with alcohol or rose-water, was the form in which it was employed. The latter was preferred where it was feared the alcohol might produce too great a degree of irritation.

---

*Lotion for Dartres.*—M. LISFRANC has lately used, with success, in cases of obstinate dartre, a lotion composed of a solution of the Chloride of Soda. He first subdues the symptoms of inflammation, and then uses the lotion.

---

### MIDWIFERY.

*Action of Borax on the Uterus.*—Two German physicians, BURDACH and WIGAND, have recommended borax in difficult labour, in consequence of which Dr. VAN KRANENDONK, of Delft, has been induced to use it in numerous cases, in most of which he has found the pains become more regular and more powerful under its exhibition; but he does not feel satisfied that this effect was always caused by the remedy in question. The Editors of the work in which his observations are detailed (*Tijdschrift-voor Genees verlos en Scheikundige Wetenschappen*,) have appended a note, in which they state that, for some time back, there has been a prodigious consumption of borax among the quacks in Holland; and they recommend great caution in experimenting with this medicine.

---

*Accouchement after the Death of the Mother.*—Dr. KLAATSCH, of Berlin, has related (in the *Zeitschrift für die Staatsarzneikunde*,) the case of a pregnant female, who was supposed to have been poisoned by her husband with arsenic, in consequence of which suspicion the body was disinterred a month after it had been buried. The fact of her having been poisoned was thus ascertained, and at the same time the phenomenon was discovered of a fœtus, about the seventh month, lying between the thighs of the woman; the accouchement having taken place after her death. To explain this circumstance, and similar cases related by various authors, Dr. Klaatsch supposes that the extrication of a quantity of gas in the intestines becomes a mechanical cause of the expulsion of the fœtus, accompanied in general with inversion of the uterus, which is facilitated by the complete state of relaxation of that organ. The same opinion was advanced by M. Deneux, in a Memoir on this subject published in 1822.

---

*Secale Cornutum.*—M. Goupil has lately published an interesting paper upon the powers of this medicine. He has brought together the opinions of practitioners of various countries, and from all the testimony he has collected the following inferences are drawn:

The secale cornutum excites uterine contraction during labour.

We may, without apprehension, administer two drachms and a half, if not at one dose, at least in divided doses in the course of a few hours.

The uterine contractions produced by this remedy are usually manifested by pains in the lumbar region, which gradually become quicker, and are at length either constant or remittent. After some time they cease entirely.

The action of this medicine, given in moderate doses, is quick; it continues from one to three hours, and may be renewed by a repetition of the dose.

We cannot, with propriety, administer the secale cornutum when delivery is obstructed by any mechanical obstacle. It is only useful when increased uterine contractions are required.

It does not augment the rigidity of the neck of the uterus, and may be given before that part is dilated.

It is not only useful in hastening delivery in cases of inertia of the uterus; but it is equally serviceable in expelling retained placentæ, in checking hemorrhage which depends upon the non-contraction of the uterus, by causing a discharge of the clots of blood with which that viscus may be filled.

When the secale cornutum has been given during labour, hemorrhage very rarely takes place after delivery.

The diminution of the lochiæ, which has been mentioned in several works as one effect of this substance, has been observed but in a very few cases.

The secale cornutum does not produce, in delicate and nervous women, those bad effects which have been apprehended, from the statements of some writers.

As it is capable of provoking abortion, it should never be given without the sanction of a medical practitioner.

To obtain the full effects of the secale cornutum, the following directions must be attended to:—It should be used the same year it is collected. It must be kept in a well-closed bottle, and should neither be bruised nor powdered until the moment it is to be employed. The powder is the most efficacious form in which this medicine can be given. (*Journal des Progres des Sciences.*)

---

### PHARMACY.

*Extraction of Morphia from dry Poppy Heads;* by M. Tilloy.—Make an aqueous extract of the heads, add alcohol to the extract, separate the alcoholic solution, and distil it; by this means the gummy matter is separated. An extract like syrup will be obtained by the distillation, which, being heated to make it thinner, and of the consistency of treacle, is to be again treated with alcohol; a separation of more gum, with much nitrate of potash, will be effected. The solution being withdrawn, is to be distilled, and the extract which will remain is to be acted upon by a sufficient quantity of water, and filtered, to separate the resinous matter present. The morphia may then be separated from this liquid, either by ammonia, carbonate of soda, or magnesia. Ammonia does not precipitate all the morphia; carbonate of soda precipitates a large quantity, but it separates resinous matter also, which is found mingled with the morphia. Magnesia is preferable; but, as the liquid contains much free acetic acid, it is expensive to employ the necessary quantity of pure magnesia: the liquid may therefore be partly saturated, whilst hot, by carbonate of magnesia, or even by carbonate of lime. A judgment, when no more must be added, must be formed from the effervescence; then pure magnesia is to be added, which will cause the liberation of ammonia; the whole is to be left for twenty-four hours to cool; being then filtered, the precipitate is to be washed, and, when dry, acted upon by alcohol. Operating in this manner, morphia may be obtained from all kinds of poppies. (*Journal of Science*, from the *Bull. Univ.*)

---

### MISCELLANEOUS.

*Professional Fees.* (From Mr. WADD's *Mems. Maxims, and Memoirs.*)

Much curious material might be collected illustrative of individual and national notions, both in ancient and modern times, as to the "quantum meruit," or honorarium, to professional men.

The Chinese proceed on the principle of "no cure no pay:" the termination of the case, therefore, decides the fee; except in court practice, where a very effectual method is taken of stimulating professional skill, by stopping the salary during a royal indisposition.

In Portugal, it is no uncommon thing to pay the doctor by presenting him his own portrait. Mr. T., an English surgeon, who cured a servant of the royal family of a dangerous wound in the abdomen, was rewarded by having his picture hung up in Lapa church, standing by the patient's bed, with the Virgin Mary above, who had enabled him to perform the cure. (*Southey's Spain.*) Occasionally, however, the faculty have met with rougher treatment: thus Austrigilda, wife of Gontram king of Burgundy, had, in compliance with her dying request, her two physicians slain, and buried with her. These were probably the only two medical gentlemen who were ever privileged to lie in the tombs of kings. On the other hand, on the death of Pope Adrian, the night after his decease, the door of his chief physician was ornamented with garlands, with the inscription, "To the deliverer of his country."

The following are some of the most ancient fees on record:

Erasistratus had 60,000 crowns from Seleucus, for discovering the disorder of his son Antiochus.

Alcon, celebrated by Martial for his dexterity in curing hernias by incision, was no less nobly remunerated by the public, who repaid him, in the course of a few years' practice, ten millions of sesterces, which he had lost by a law-suit.

Aruntius, Calpetanus, Rubrius, and Albutius, for their attendance on the Emperor Augustus, and his two immediate successors, had each an annual salary of two hundred and fifty thousand sesterces, equal to two thousand pounds of English money.

Petrus Aponensis, a physician of Padua, in the thirteenth century, we find refusing to go out of the town to see a patient, under 150 francs a-day. When sent for to Pope Honorarius IV., he demanded 400 ducats a-day. (*Vander Linden de Scriptoribus Medicis.*)

The mode and manner of remunerating medical services in the early periods of our own country, we may learn from the following items:

1345. Edward III. granted a pension of sixpence a-day to his apothecary, Coursus de Gungeland. And "Ricardus Wye, chirurgicus, habet duodecim denarios per diem et octo marcas per annum, pro vadiis suis pro vita."

Pet. 45. Ed. III. "Quod Willielmus Holme Sirurgicus Regis pro vita sua possit, fugare, capere, et asportare omnimodas feras in quibus cunque forestis, chaccis, parcis et warrennis regis"

In the court of the kings of Wales, the physician, or surgeon, was the twelfth person in rank, and appears to have had by law certain established fees. For curing a flesh-wound that was not dangerous, he was allowed no other perquisite than such of the garments of the wounded person as were stained with blood; but, for curing any of the three dangerous or mortal wounds, he was allowed a fee of 180 pence, and his maintenance, besides the blood-stained garments.

Among the expences of the Earl of Cumberland, in the seventeenth year of Henry VIII., we have "Item, in a reward paid by my lord to a physician of Cambridge, the 17th of June, £1." "Item to a physician at Westminster, for seying my lord's water, iv*d*." (*Whitaker's Craven*, p. 233)

In Burn's History of Westmorland, vol. 1, p. 99, is a curious indenture, between Sir Walter Strickland, knt. and a doctor of physic, who was to have £20. to cure him of an asthma.—18th year of Henry VIII.

Our physicians, says Stow, are allowed to be men of skill in their profession, and well versed in other parts of learning. We do not often suffer by their ignorance; but the great grievance here is, that the inferior people are undone by the exorbitance of their fees. Half a crown is looked upon as a great fee in Holland. A physician scorns to touch any metal but gold with us, and our surgeons are still more unreasonable. (*Stow*, vol. ii. p. 558.)

In a book called "Levamen Infirmi," the usual fees to physicians and chirurgeons at that time (1700,) are thus stated:—"To a graduate in physic, his due is about ten shillings, though he commonly expects or demands twenty. Those that are only licensed physicians, their due is no more than six shillings and eight pence, though they commonly demand ten shillings. A surgeon's fee is twelve pence a mile, be his journey far or near; ten groats to set a bone broke, or out of joint; and for letting blood, one shilling; the cutting off, or amputation, of any limb, is five pounds; but there is no settled price for the cure."

In modern times (1737,) we find the physicians who attended Queen Caroline had 500 guineas, and the surgeons 300 guineas, each.

Dr. Willis, for his successful attendance on his Majesty King George III., was rewarded by £1500. per annum for twenty years, and £650. per annum to his son for life. The other physicians had thirty guineas each visit to Windsor, and ten guineas each visit to Kew.

Baron Dimsdale, for his inoculation of the Empress of Russia and her son, was made a baron of the empire, with a present of £12,000., and a pension of £500. per annum.

Sir Theodore Mayerne, who got an immense sum by his practice, was once consulted by a friend, who laid two broad pieces of gold upon the table, (six and thirties,) and Sir Theodore put them into his pocket. The friend was hurt at his pocketing such a fee, but Sir Theodore said to him, "I made my will this morning, and, if it should appear that I had refused a fee, I might be deemed *non compos.*"—The late Mr. Martin, the surgeon, knew a modern doctor who improved this practical joke. When he was a young man, he sometimes went to Dr. Meyer Schomberg's, who was much resorted to. Martin was shown in to him one morning while he had a patient with him: when the patient was gone, Martin observed two guineas lying on the table; he asked the Doctor how he happened to leave his money about in that manner? "I always have a couple of guineas before me," said the Doctor, "as an example, or broad hint, what they ought to give."

